# miRNA-145 inhibits VSMC proliferation by targeting CD40

**DOI:** 10.1038/srep35302

**Published:** 2016-10-12

**Authors:** Xin Guo, Dai Li, Min Chen, Lei Chen, Bikui Zhang, Tian Wu, Ren Guo

**Affiliations:** 1 Department of Pharmacy, The Third Xiangya Hospital, Central South University, Changsha, Hunan, China; 2 National Institution of Drug Clinical Trial, Xiangya Hospital, Central South University, Changsha, Hunan, China; 3 Department of Pharmacy, The Second Xiangya Hospital, Central South University, Changsha, Hunan, China

## Abstract

Recent studies have demonstrated functions of miR-145 in vascular smooth muscle cells (VSMCs) phenotypes and vascular diseases. In this study, we aim to determine whether CD40 is involved in miR-145 mediated switch of VSMC phenotypes. In cultured VSMCs, the effects of miR-145 and CD40 on TNF-α, TGF-β, and Homocysteine (Hcy) induced cell proliferation were evaluated by over-expression of miR-145 or by siRNA-mediated knockdown of CD40. We also used ultrasound imaging to explore the effect of miR-145 on carotid artery intima-media thickness (CIMT) in atherosclerotic cerebral infarction (ACI) patients. The results showed 50 ng/mL TNF-α, 5 ng/mL TGF-β, and 500 μmol/L Hcy significantly increased the expression of CD40, both at mRNA and protein levels, and also induced the proliferation of VSMCs. We found that over-expression of miR-145 significantly inhibited the expression of CD40 and the differentiation of VSMCs, and over-expression of miR-145 decreased IL-6 levels in VSMC supernatants. In ACI patients, the lower expression of miR-145 was associated with thicker CIMT and higher levels of plasma IL-6. Our results suggest that the miR-145/CD40 pathway is involved in regulating VSMC phenotypes in TNF-α, TGF-β, and Hcy induced VSMCs proliferation model. Targeting miR-145/CD40 might be a useful strategy for treating atherosclerosis.

Vascular smooth muscle cells (VSMCs) are the main cell type in blood vessel walls, where they play an important role in maintaining the normal physiological function of blood vessels. An increasing number of studies suggest that, in the different states of atherosclerosis (AS), the structure and function of VSMCs in atherosclerotic plaques are dynamically changed. Additionally, VSMCs in bloods vessel walls switch between differentiated and dedifferentiated phenotypes is required during vascular remodeling in response to injury, including blood vessel damage, mechanical or blood dynamic stress, and inflammation.

MicroRNAs (miRNAs) are 21- to 22-nucleotide noncoding small RNAs^1^,^2^, that play a negative role in regulating their target mRNA by binding the miRNA regulatory elements located in the 3′-untranslated region (3′-UTR) of the mRNAs^3^. Growing evidence suggests miRNAs play important roles in cellular processes such as proliferation, apoptosis and differentiation^4^,^5^. Recent studies suggest that the switch of VSMC phenotypes is regulated by miRNAs, particularly under different disease states. MiR-145 is the most abundant miRNA in the vascular wall^6^, and the functions and regulation of miR-145 in vascular disease have been the focus of many recent studies^7^,^8^,^9^. miR-145 serves as a VSMC phenotypic marker and modulator in controlling vascular neointimal lesion formation; the mechanism involves Kruppel-like factor (KLF) 5 and the downstream signaling molecule, myocardin. However, a certain miRNA may exert its influence on more than one target gene, and it is believed that miR-145 is involved in different diseases via different pathways.

CD40 is a 50-kDa type I transmembrane glycoprotein receptor that is expressed in numerous cell types, such as VSMCs, endothelial cells (ECs), platelets, fibroblasts, and immunity cells^10^. CD40 belongs to the tumor necrosis factor superfamily^11^, and the activation of CD40 by its transmembrane ligand CD40L leads to the up-regulation of many proinflammatory and proatherogenic genes via multiple downstream signaling pathways^12^. Aberrant CD40/CD40L signaling in the vasculature is associated with atherosclerosis, myocardial infarction, and Kawasaki disease^13^,^14^,^15^,^16^. Additionally, our previous research also revealed that a SNP in the CD40 gene (rs1883832) is associated with an increased risk of ischemic stroke and post-stroke epilepsy^17^,^18^. However, there is limited research on the interaction of CD40 and miRNAs in regulating VSMC phenotypes.

In this study, we first explored the association of miR-145 expression with carotid artery intima-media thickness (CIMT) in patients with or without atherosclerosis cerebral infarction (ACI); then, we defined the role of miR-145 in CD40 expression both *in vivo* and *in vitro*. Additionally, we explored the regulation of CD40 by miR-145 in the switch of VSMC phenotypes. Thus, the aims of this research study were to identify a novel pathway that mediates VSMC phenotypes and to propose a new therapeutic strategy for ACI.

## Results

### CD40 signaling is involved in the switch of VSMC phenotype

**Figure 1:**
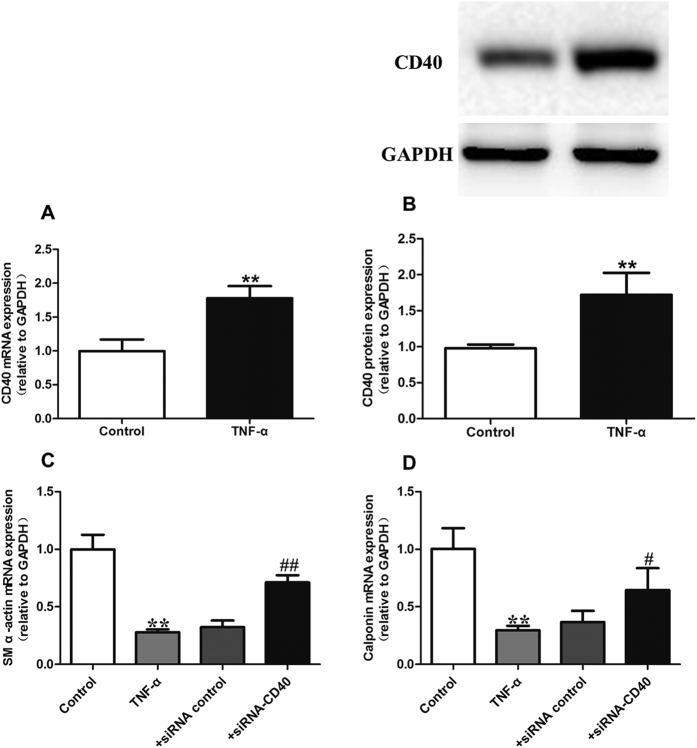
TNF-α induced proliferation model of VSMCs in a CD40 dependent pathway. (**A**) CD40 mRNA expression after treating with 50 ng/mL TNF-α in VSMCs; (**B**) CD40 protein expression after treating with 50 ng/mL TNF-α in VSMCs; (**C**) SM α-actin mRNA expression in each group; (**D**) Calponin mRNA expression in each group. All values are expressed as the mean ± SD, n = 3. **P < 0.01, compared with Control group, ^#^*P* < 0.05, ^##^*P* < 0.01, compared with TNF-α group.

We used a TNF-α induced proliferation model of VSMCs to detect the role of CD40 signaling in the regulation of the VSMC phenotype. As shown in Fig. 1A,B, treatment of VSMCs with 50 ng/mL TNF-α significantly increased the expression of CD40 both at the mRNA and protein levels. We also found a suppression of the mRNA levels of VSMC differentiation marker genes such as SM α-actin and calponin. Inhibition of CD40 using the siRNA-CD40 markedly increased the SM α-actin and calponin levels in TNF-α treated VSMCs (Fig. 1C,D). These data indicate the TNF-α-mediated VSMC proliferation requires, at least in part, the induction of CD40.

### miR-145 inhibits CD40 expression at the mRNA and protein level

**Figure 2:**
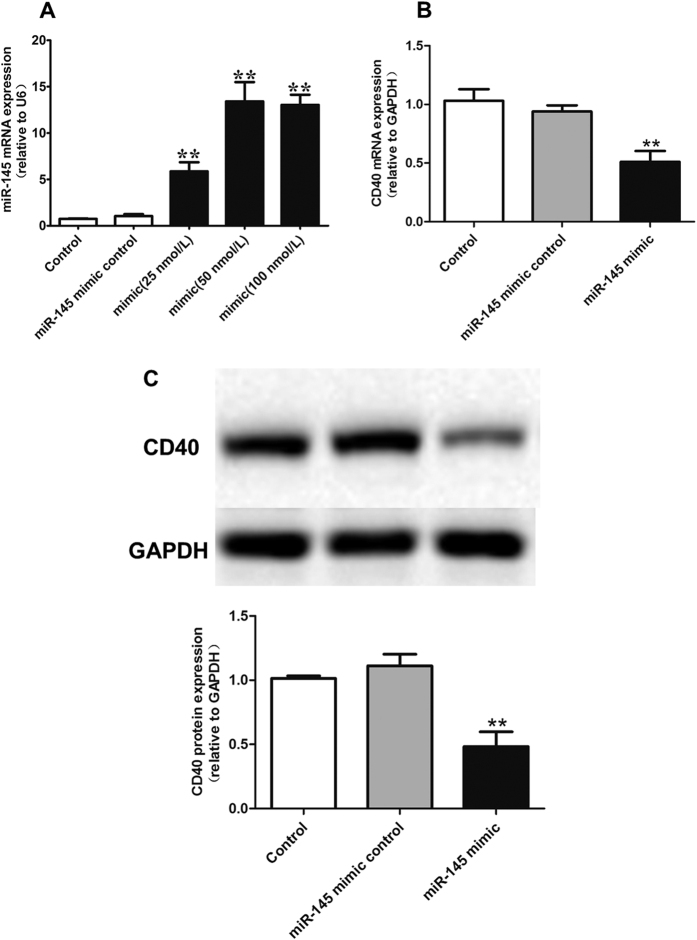
Effect of miR-145 mimic on CD40 expression. (**A**) miR-145 expression after transfection with miR-145 mimic control, 25 nmol/L, 50 nmol/L and 100 nmol/L miR-145 mimics in VSMCs; (**B**) CD40 mRNA expression after treating with miR-145 mimics; (**C**) CD40 protein expression after treating with miR-145 mimics. All values are expressed as the mean ± SD, n = 3. **P < 0.01 vs Control.

**Figure 3:**
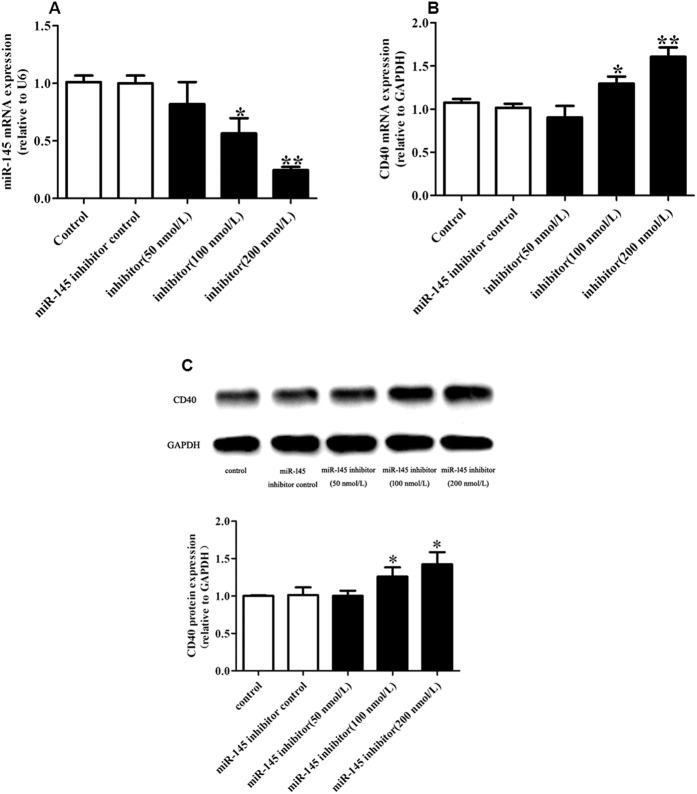
Effect of miR-145 inhibitor on CD40 expression. (**A**) miR-145 expression after transfection with miR-145 inhibitor control, 50 nmol/L, 100 nmol/L and 200 nmol/L miR-145 inhibitors in VSMCs; (**B**) CD40 mRNA expression after treating with miR-145 inhibitors; (**C**) CD40 protein expression after treating with miR-145 inhibitors. All values are expressed as the mean ± SD, n = 3. *P < 0.05, **P < 0.01 vs Control.

Using Pictar and Targetscan online software, we found that the CD40 3′-UTR contains one putative miR-145 target site, which binds with imperfect complementation to the seed sequence. To examine the negative regulation of miR-145 on CD40 in VSMCs, we transfected VSMCs with miR-145 mimics for 24 h. A dose response analysis allowed us to determine that 50 nmol/L miR-145 mimics resulted in an approximately 12-fold increase in miR-145, which was appropriate for our subsequent experiment (Fig. 2A). We then confirmed that miR-145 mimics significantly decreased CD40 expression both at the mRNA and protein level in VSMCs (Fig. 2B,C). Similarly, we have confirmed that 200 nmol/L miR-145 inhibitors was appropriate for next loss of function experiments, which can cause an approximately 4-fold decrease in miR-145 and significant up-regulation of CD40 in VSMCs (Fig. 3A–C). These data suggest that CD40 is a potential target of miR-145.

### miR-145 inhibits VSMC differentiation by suppression of CD40

**Figure 4:**
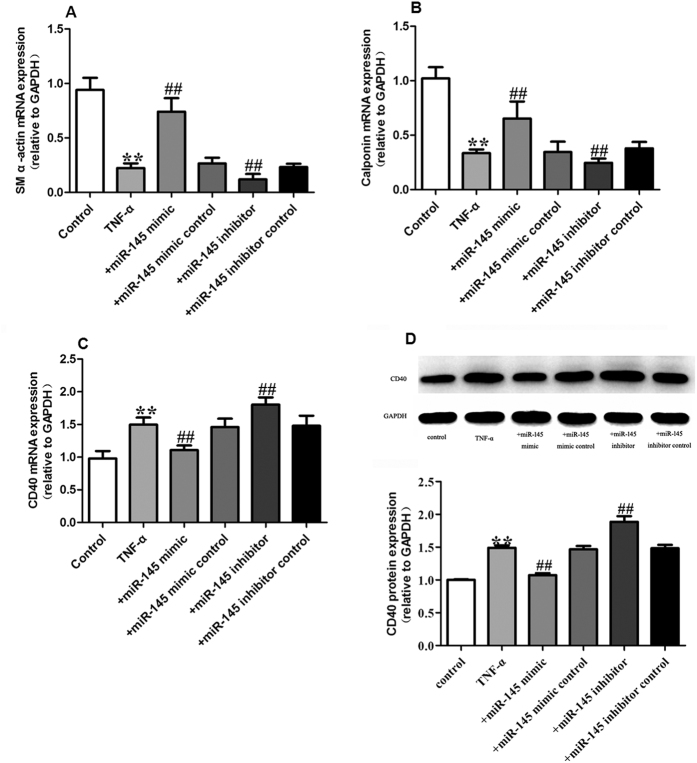
miR-145 inhibits VSMC differentiation induced by TNF-α (**A**) SM α-actin mRNA level in each group; (**B**) Calponin mRNA level in each group; (**C**) CD40 mRNA expression in each group; (**D**) CD40 protein expression in each group. All values are expressed as the mean ± SD, n = 3. **P < 0.01, compared with Control group, ^##^*P* < 0.01, compared with TNF-α group.

**Figure 5:**
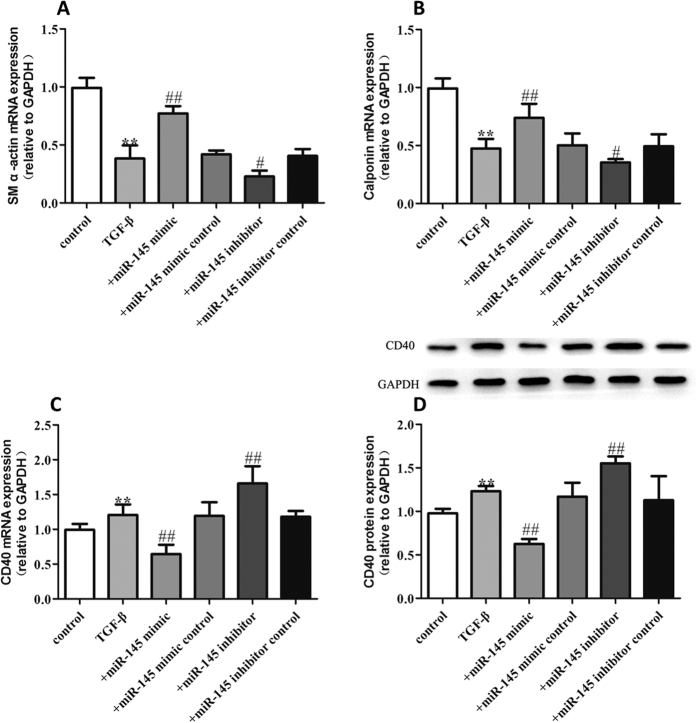
miR-145 inhibits VSMC differentiation induced by TGF-β (**A**) SM α-actin mRNA level in each group; (**B**) Calponin mRNA level in each group; (**C**) CD40 mRNA expression in each group; (**D**) CD40 protein expression in each group. All values are expressed as the mean ± SD, n = 3. **P < 0.01, compared with Control group, ^#^*P* < 0.05, ^##^*P* < 0.01, compared with TGF-β group.

**Figure 6:**
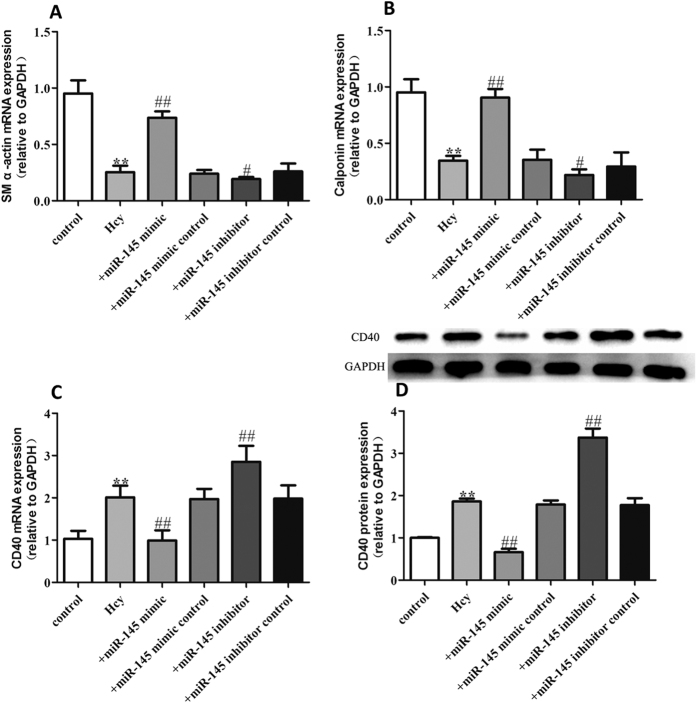
miR-145 inhibits VSMC differentiation induced by Hcy (**A**) SM α-actin mRNA level in each group; (**B**) Calponin mRNA level in each group; (**C**) CD40 mRNA expression in each group; (**D**) CD40 protein expression in each group. All values are expressed as the mean ± SD, n = 3. **P < 0.01, compared with Control group, ^#^*P* < 0.05, ^##^*P* < 0.01, compared with Hcy group.

In our study, we first determined that CD40 expression enhanced the dedifferentiated state of VSMCs. To gain further insight into the role of miR-145 in regulating TNF-α induced proliferation of VSMCs, we pretreated VSMCs with miR-145 mimics and miR-145 inhibitors 30 min before TNF-α administration. As shown in Fig. 4A,B, treatment of VSMCs with 50 ng/mL TNF-α significantly decreased VSMC differentiation marker gene expression such as SM α-actin and calponin. We found that 50 ng/mL TNF-α also induced increased expression of CD40 both at the mRNA and protein level (Fig. 4C,D). The over-expression of miR-145 through use of miR-145 mimics partially restored the expression of SM α-actin and calponin, accompanied by a decreased level of CD40 while the miR145 inhibitor caused an opposite effects on SM α-actin, calponin and CD40 (Fig. 4A–D). Compared to the TNF-α group, the miR-145 mimic control and miR-145 inhibitor control did not show any significant effect on the expression of SM α-actin and calponin. In addition to TNF-α, there are some other stimuli that were well established to induce VSMCs proliferation. So in this study, we also tested the role of miR-145/CD40 pathway on TGF-β and Homocysteine (Hcy) induced VSMCs proliferation model. As shown in Fig. 5 and 6, in line with the TNF-α treated model, 5 ng/mL TGF-β, and 500 μmol/L Hcy both induced increased expression of CD40 accompanied by decreased levels of SM α-actin and calponin. miR-145 mimics partially reversed the expression of SM α-actin, calponin and CD40 induced by TGF-β and Hcy while the miR145 inhibitor caused an opposite effects on SM α-actin, calponin and CD40. Together, our data suggest that miR-145 contributes to the contractile phenotype of VSMC partially by blocking the increased expression of CD40.

### The effects of over-expression and inhibition of miR-145 on the EdU positive numbers of VSMCs induced by TNF-α, TGF-β and Hcy

**Figure 7:**
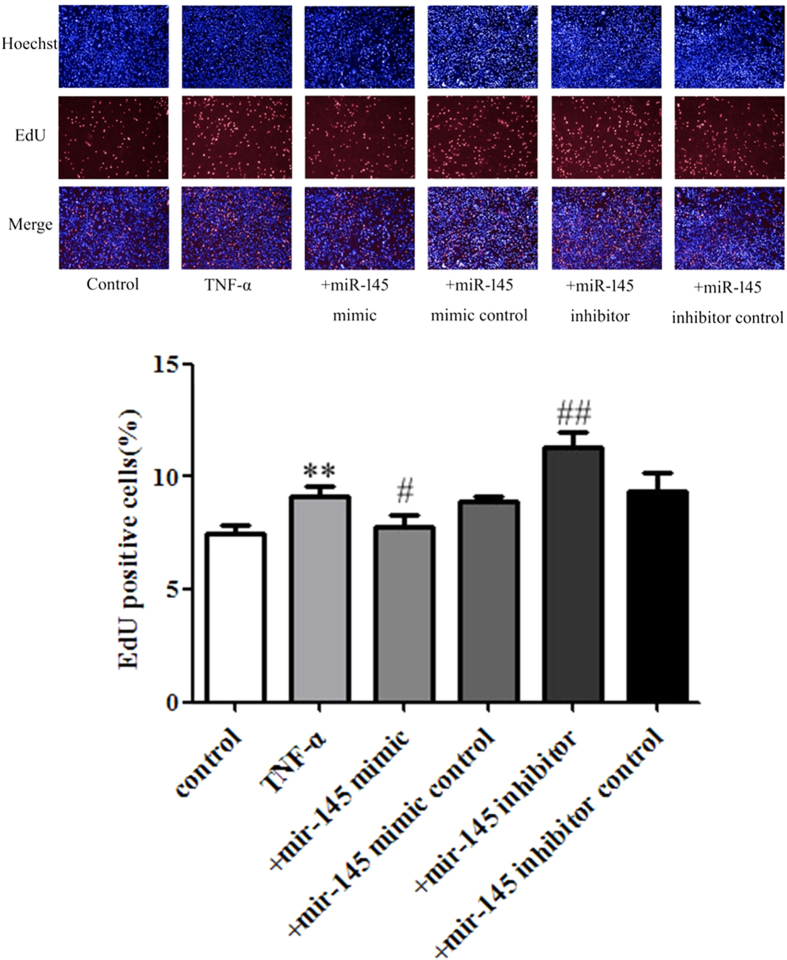
The effects of over-expression and inhibition of miR-145 on the EdU positive numbers of VSMCs induced by TNF-α The EdU positive VSMCs numbers in each group. All values are expressed as the mean ± SD, n = 3. **P < 0.01, compared with Control group, ^#^*P* < 0.05, ^##^*P* < 0.01, compared with TNF-α group.

**Figure 8:**
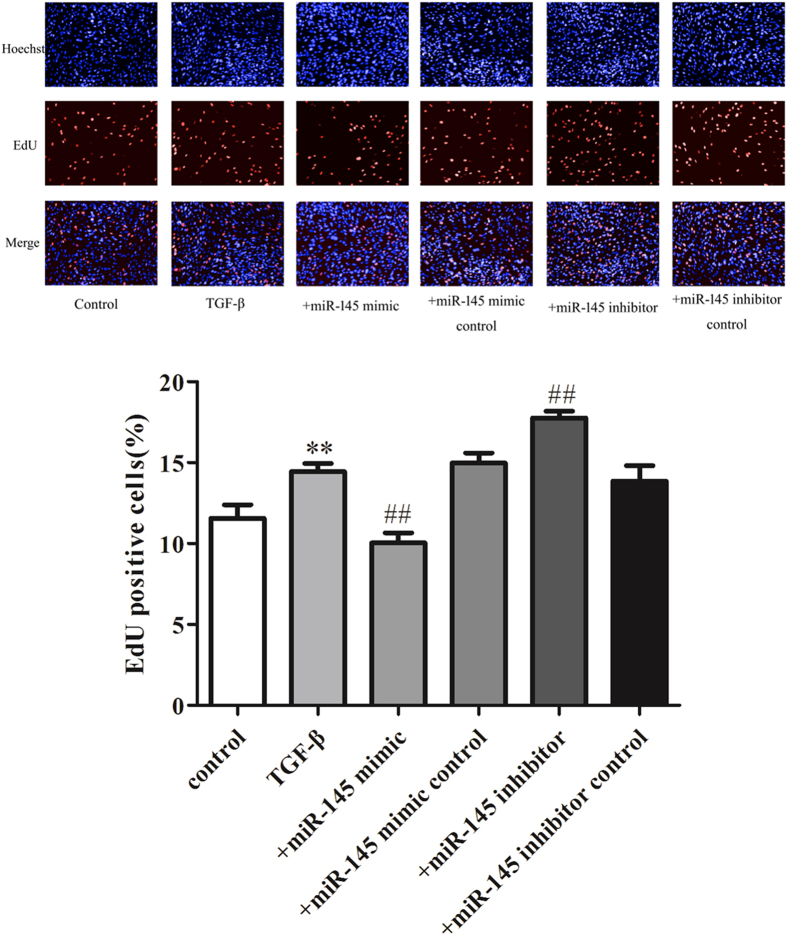
The effects of over-expression and inhibition of miR-145 on the EdU positive numbers of VSMCs induced by TGF-β The EdU positive VSMCs numbers in each group. All values are expressed as the mean ± SD, n = 3. **P < 0.01, compared with Control group, ^##^*P* < 0.01, compared with TGF-β group.

**Figure 9:**
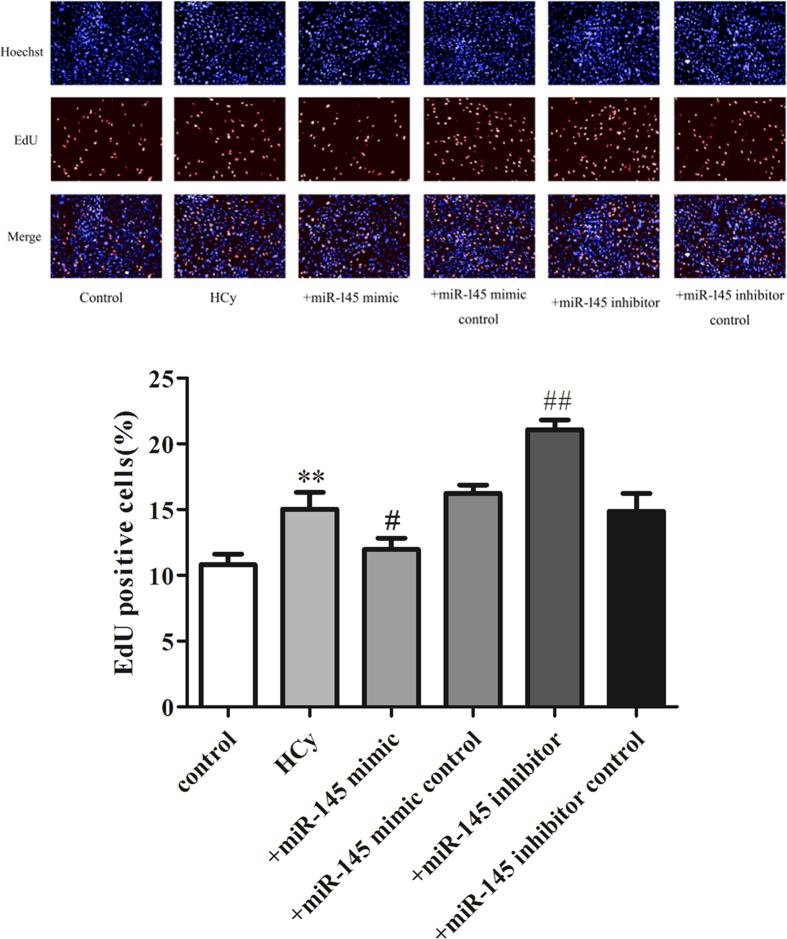
The effects of over-expression and inhibition of miR-145 on the EdU positive numbers of VSMCs induced by Hcy The EdU positive VSMCs numbers in each group. All values are expressed as the mean ± SD, n = 3. **P < 0.01, compared with Control group, ^#^*P* < 0.05, ^##^*P* < 0.01, compared with Hcy group.

EdU (5-Ethynyl-2′-deoxyuridine) is a type of thymidine nucleoside analogue that can be assembled into DNA molecules by replacing thymine (T) during DNA replication. Based on the specific reaction between Apollo^®^ fluorescent dyes and the EdU, DNA replication can be directly and accurately detected, and so EdU staining is widely used in the detection of proliferation. As shown in Fig. 7, treatment of VSMCs with 50 ng/mL TNF-α significantly increased the EdU positive cell numbers. The over-expression of miR-145 by miR-145 mimics partially decreased the EdU positive VSMC numbers while the miR-145 inhibitor increased the EdU positive VSMC numbers induced by TNF-α. Furthermore, 5 ng/mL TGF-β and 500 μmol/L Hcy also caused dramatically increased EdU positive cells. Pretreatment of VSMC with miR-145 mimics before TGF-β and Hcy administration significantly decreased the EdU positive VSMC numbers while the miR-145 inhibitor increased the EdU positive VSMC numbers (Figs 8 and 9). Compared to the model group, the miR-145 mimic control and miR-145 inhibitor control treatments did not show any significant effect on EdU positive cell numbers. Because miR-145 serves as a negative regulator of CD40, these data indicate that miR-145 suppresses CD40, thereby reducing the EdU positive cell numbers.

### Effect of miR-145 on sCD40L and IL-6

**Figure 10:**
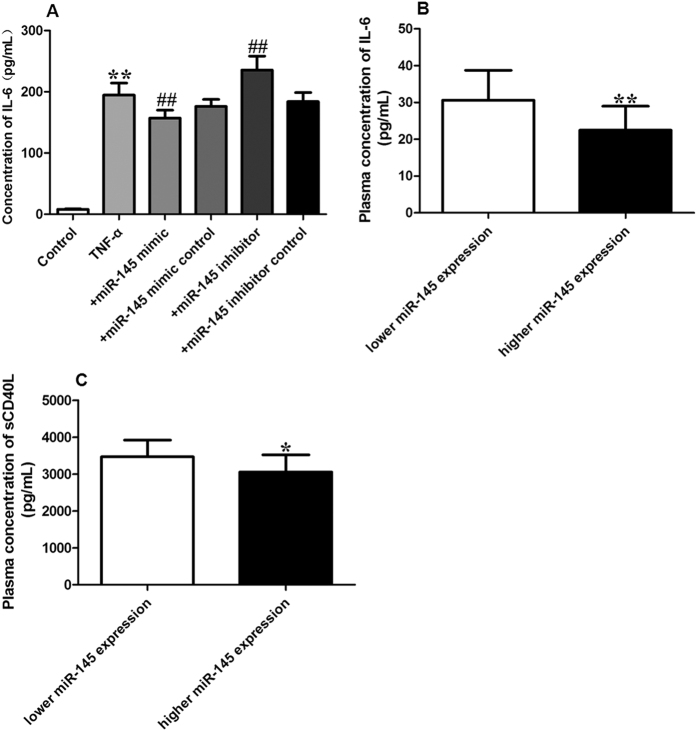
Effect of miR-145 on sCD40 L and IL-6 (**A**) The concentration of IL-6 in each group of VSMCs (n = 3); (**B**) Plasma concentration of IL-6 in ACI patients with lower or higher expression of miR-145 (n = 120); (**C**) Plasma concentration of sCD40 L in ACI patients with lower or higher expression of miR-145 (n = 120). All values are expressed as the mean ± SD. *P < 0.05, **P < 0.01, compared with Control group or lower miR-145 expression group, ^##^*P* < 0.01, compared with TNF-α group.

Numerous studies have reported the vital role of CD40/CD40L system in inflammation associated diseases^19^,^20^,^21^. To demonstrate whether miR-145 regulates the inflammation level in VSMCs treated with TNF-α and in ACI patients, we collected the supernatants from VSMCs and the plasma from ACI patients to detect the concentration of IL-6 and sCD40L. As shown in Fig. 10A, significant increases in IL-6 levels were observed in TNF-α treated VSMCs. Over-expression of miR-145 by miR-145 mimics significantly decreased the levels of IL-6 while miR-145 inhibitors significantly increased the levels of IL-6 induced by TNF-α. In line with the *in vitro* experiment, we also found increased levels of IL-6 and sCD40L in ACI patients with lower expression of miR-145 (Fig. 10B,C). These data suggest that miR-145 is involved in the process of inflammation by suppressing the levels of sCD40L and IL-6.

### Effect of miR-145 on CIMT

**Figure 11:**
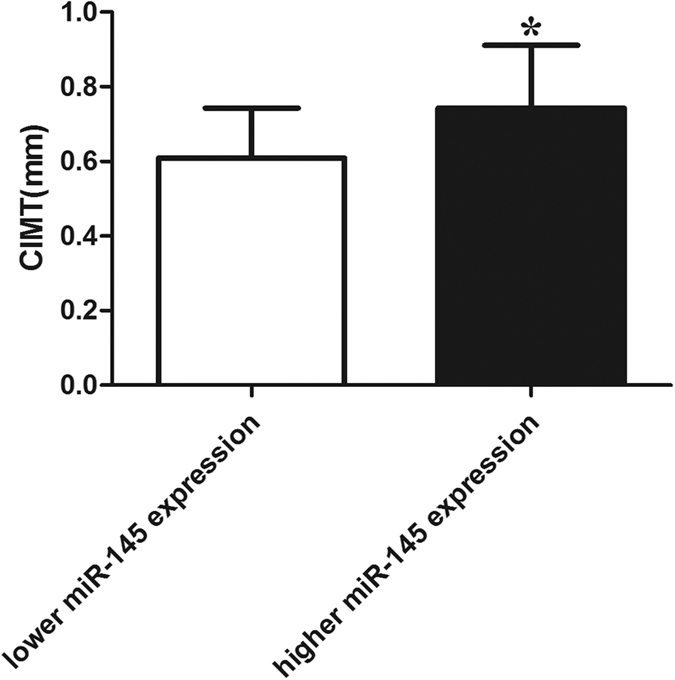
Effect of miR-145 on CIMT The mean CIMT in the patients with lower or higher expression of miR-145. All values are expressed as the mean ± SD, n = 50. *P < 0.05, compared with lower miR-145 expression group.

CIMT evaluation by ultrasound has become an easy, safe, non-invasive method to detect subclinical atherosclerosis and has been shown to be an effective predictor of cardiovascular events^22^,^23^. Current studies have indicated an important role of miR-145 in the process of atherosclerosis both in animal models and patients^24^,^25^,^26^. Therefore, in our study, we explored the relationship between CIMT and miR-145 level in ACI patients. According to the miR-145 expression level (median: 1.40; data range: 0.32~2.46) in ACI patients, we divided the patients into two groups: those with miR-145 level higher than the median and those with miR-145 level lower than the median; the demographic characteristics of the subjects are shown in Table 1. As shown in Fig. 11, we found that the mean CIMT in patients with lower expression of miR-145 was significantly greater than those with higher expression of miR-145. These data suggest a definite correlation between CIMT and miR-145; miR-145 may also be a potential biomarker for atherosclerosis.

### miR-145 directly target CD40 in VSMCs

**Figure 12:**
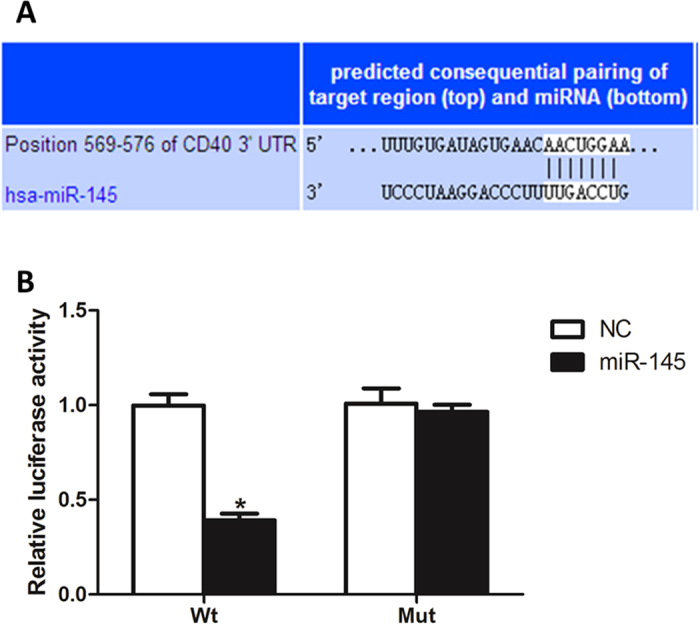
Dual luciferase reporter assay (**A**) The potential miR-145 binding sequence of CD40 3′-UTR. (**B**) Luciferase activities of wild-type 3′-UTR CD40 and mutant 3′-UTR CD40 constructs in VSMCs. All values are expressed as the mean ± SD, n = 3. *P < 0.05, compared with negative control group.

TargetScan 6.2 was used to predict the potential target of miR-145. CD40, which is known to serve a critical role in the process of atherosclerosis was predicted as one of the targets (Fig. 12A). Luciferase activities assay has shown that miR-145 significantly inhibited the luciferase activity of wild-type but not the mutant 3′-UTR of the CD40 gene in VSMCs (Fig. 12B). The result suggests that CD40 is a direct target of miR-145.

## Discussion

miRNAs regulate gene expression at the post-transcriptional level and are associated with various cardiovascular diseases. Our study identified miR-145/CD40 as a novel pathway in regulating the VSMC phenotype and also explored the role of this pathway in the process of ACI. Our data support that the level of miR-145 was significantly lower in ACI patients than in the controls; we also observed that decreased miR-145 levels were accompanied by increased CD40 levels. Therefore, we propose that targeting of the miR-145/CD40 pathway may be an effective therapeutic strategy for ACI.

Activation of the CD40/CD40L pathway leads to the up-regulation of many proinflammatory and proatherogenic genes, which is an important process in many diseases, particularly in atherosclerotic diseases^27^. Increased CD40 levels are found in atheroma-associated cells including ECs, VSMCs, and macrophagocytes^28^,^29^. Inhibition of CD40 via gene knockout or antibody can significantly reduce the volume of AS plaques in mice, accompanied by increased plaque stability and decreased risk for cardio-cerebral vascular accidents. *In vitro* studies have also shown that CD40/CD40L interactions lead to VSMC activation and subsequent adhesion molecule expression, which is a vital process in the initiating step of AS^29^. However, there are limited studies on the role and regulating mechanism of CD40 on VSMC phenotype. Consistent with the important role of CD40 in AS, the data in our study indicate CD40 was significantly up-regulated in the TNF-α, TGF-β and Hcy induced VSMC proliferation model. Inhibition of CD40 by CD40-siRNA resulted in increased expression of VSMC differentiation marker genes such as SM α-actin and calponin, which indicated the contractile phenotype of VSMC. Additionally, we also observed a decreased level of miR-145 accompanied by the up-regulation of CD40 in VSMCs. By using Pictar and Targetscan online software, we found CD40 is a potential target gene of miR-145, which prompted us to investigate whether miR-145 regulates the VSMC phenotype though targeting of CD40.

Early research on miR-145 mainly focused on its association with the occurrence and development of cancer. It is reported that altered expression of miR-145 is associated with colorectal, breast, and prostate cancer^30^,^31^,^32^. Recent studies have identified that miR-145 is the most abundant miRNA in vascular walls and in freshly isolated VSMCs^33^. Subsequent research studies have demonstrated miR-145 as a novel VSMC phenotypic marker and modulator, as well as the involvement of miR-145 in vascular neointimal lesion formation via KLF5 and its downstream signaling molecule, myocardin^34^. One specific miRNA can repress multiple target mRNAs by imperfect base-pairing between the miRNA and target mRNAs, so there may be other miR-145 associated pathways involved in the regulation of the VSMC phenotype. CD40 serves as a link between inflammation, atherosclerosis and thrombosis; our functional tests have successfully identified miR-145 as being involved in the regulation of CD40 because over-expression of miR-145 significantly inhibits the expression of CD40. In further *in vitro* studies, our results also showed that over-expression of miR-145 via miR-145 mimics could result in a differentiated state of VSMCs, as reflected by a decrease in the expression of CD40 and an increase in the expression of SM-actin and calponin. In contrast, suppression of miR-145 via specific inhibitors could result in a dedifferentiated state of VSMCs, as reflected by the increased expression of CD40 and decreased levels of SM α-actin and calponin. In order to directly observe the proliferation of VSMCs in different treatments, we also used EdU staining to count the proliferative VSMCs. As expected, over-expression of miR-145 via miR-145 mimics significantly reduced the proliferative cell numbers of VSMCs induced by TNF-α, TGF-β and Hcy, the expression of CD40 was also down-regulated in these cells. Furthermore, in the loss of function experiments, inhibition of miR-145 by miR-145 inhibitors caused an opposite effect in comparison with miR-145 mimics in VSMCs. This suggests that the effect of miR-145 in regulating the VSMC phenotype partially depends on CD40.

Our previous study has already demonstrated an increase in CD40 expression in ACI patients^35^, and the data in this study further prove the involvement of miR-145 in ACI patients. We observed that the mean CIMT in the ACI patients with lower levels of miR-145 was significantly greater than in those with higher levels of miR-145. To our knowledge, this is the first study to explore the relationship between miR-145 levels and CIMT in ACI patients. Increased CIMT is often associated with increased risk for future coronary heart disease and stroke, and our findings have already identified a regulatory function of miR-145 on CD40, which is involved in the pathogenesis of AS; therefore, the miR-145 level may be used as a biomarker for the diagnosis and treatment of AS.

In conclusion, the present study reveals that miR-145 is decreased in patients with ACI and in proliferative VSMCs induced by TNF-α, TGF-β and Hcy. Over-expression of miR-145 negatively regulates its target gene CD40 and results in a contractile phenotype of VSMCs. However, further research focusing on the role of miR-145/CD40 pathway in inflammation or CVD/atherosclerosis model is still necessary to verify our findings and other pathogenic factors of cardiovascular diseases should also be considered in subsequent studies. The findings in this study indicate a novel pathway in which miR-145 and CD40 are involved. Our research may also have extensive implications for the diagnosis and therapy of ACI.

## Methods

### Cell culture

VSMCs were cultured in Dulbecco’s modified Eagle’s medium (DMEM, Gibco) with 10% fetal bovine serum (Gibco) in a 5% CO_2_ incubator at 37 °C. When the cell density reached more than 80%, sterilized PBS was used to wash the cells twice, then 0.25% trypsin was used to dissociate the cell-cell contacts, followed by the use of complete medium to terminate trypsinization when the edges of the cells narrowed. Cells were gently dislodged from the cell culture surfaces by a Pasteur pipet, and collected by centrifugation (1000 r/min, 5 min). After centrifugation, the cells were resuspended in complete medium with 10% fetal bovine serum for further experiments.

### Real-time PCR analysis

To validate differentially expressed CD40, SM α-actin and Calponin mRNAs were assessed on VSMCs from different groups. Real-time PCR was performed according to the manufacturer’s instructions using the ABI 7300 real-time PCR system with the SYBR Green method. Sequences of primers were as follows: CD40: 5′-GCAGGCACAAACAAG ACTGA-3′ (sense) and 5′-TCGTCGG GAAATTGATCTC-3′ (antisense); SM α-actin: 5′-GGGTGATGGTGGGAATGG-3′ (sense); 5′-GCAGGGTGGGATGCTCTT-3′ (antisense); Calponin: 5′-AACCATACACAGGTGCAGTC-3′ (sense); 5′-GATGTTC CGCCCTTCTCTTAG-3′ (antisense); GAPDH (endogenous control): 5′-CTGCACC ACCAACTGCTTAG-3′ (sense); 5′-AGGTCCACCACTGACACGTT-3′ (antisense). Relative abundance of the CD40, SM α-actin and Calponin mRNAs from VSMCs was normalized to the expression level of GAPDH. All amplification reactions were performed in triplicate.

### Measurement of IL-6 concentration

In this study, the levels of IL-6 in the cell supernatant and plasma were measured using an ELISA kit (R&D, Minneapolis, USA) following the instructions provided by the manufacturer.

### Western blotting

Protein was extracted from cultured VSMCs with RIPA lysis buffer (containing 0.1% PMSF) (Beyotime Biotech, China) following the manufacturer’s instructions. An equal amount (100 μg) of the total protein was separated by SDS-polyacrylamide gel electrophoresis (SDS-PAGE) (Beyotime Biotech, China) and then transferred onto a polyvinylidene difluoride (PVDF) (Pell, USA) membrane after electrophoresis. The membranes were immunoblotted with Abs against CD40 (human monoclonal antibody, Abcam, UK) or GAPDH (Abcam, UK) followed by a horseradish peroxidase-conjugated secondary. The immunoblots were detected by a Bio-Rad Calibrated Densitometer.

### Ultrasound Imaging

A portable ultrasound machine (SonoSite MTurbo, SonoSite, Inc., USA) was used to detect the left and right carotid arteries of ACI patients at the Third Xiangya Hospital. Optimized images of left and right carotid artery intima-media thickness (CIMT) were captured at the end of diastole. The value of the IMT on the far wall of the left and right sides in an area free of atherosclerotic plaque was recorded for further study.

### Dual luciferase reporter assay

The wild-type human CD40 3′-UTR sequence containing predicted miR-145 target sites and mutant 3′-UTR of CD40 were synthesized and inserted into the pGL3 control vector (Promega). For the reporter assay, VSMCs were seeded into 24-well plates and performed co-transfections of CD40-3′-UTR or mut-CD40-3′-UTR plasmid with miR-145 mimics or negative control. Luciferase activities were measured using a dual-luciferase reporter assay kit (Promega) according to the manufacturer’s instructions.

### Statistical analysis

SPSS software (Version 11.5) was used for the statistical analysis. The data were expressed as the mean ± SD. The differences among the groups were compared using a one-way ANOVA. *P* < 0.05 was considered to be statistically significant.

## Additional Information

**How to cite this article**: Guo, X. *et al*. miRNA-145 inhibits VSMC proliferation by targeting CD40. *Sci. Rep*. **6**, 35302; doi: 10.1038/srep35302 (2016).

## Figures and Tables

**Table 1:** General characteristics of the ACI patients.

Parameter	Low miR-145 (n = 50)	High miR-145 (n = 50)	*P*
miR-145 expression	0.90 ± 0.32	1.91 ± 0.36	<0.001
Age, year	49.65 ± 3.46	47.86 ± 3.68	NS
BMI, kg/m^2^	23.44 ± 2.04	23.98 ± 1.69	NS
SBP, mmHg	142.46 ± 7.97	139.57 ± 8.24	NS
DBP, mmHg	86.42 ± 6.53	87.38 ± 7.27	NS
Creatinine (Cr), μmol/L	85.42 ± 10.54	84.72 ± 11.47	NS
HDL-C, mmol/L	1.31 ± 0.41	1.29 ± 0.56	NS
LDL-C, mmol/L	2.71 ± 0.38	2.68 ± 0.62	NS
Triglyceride (TG), mmol/L	1.88 ± 0.35	1.90 ± 0.47	NS
Total cholesterol (TC), mmol/L	4.91 ± 0.65	4.87 ± 0.74	NS
